# COMIRESTROKE—A clinical study protocol for monitoring clinical effect and molecular biological readouts of *COM*prehensive *I*ntensive *RE*habilitation program after *STROKE*: A four-arm parallel-group randomized double blinded controlled trial with a longitudinal design

**DOI:** 10.3389/fneur.2022.954712

**Published:** 2022-11-01

**Authors:** Kamila Řasová, Patrícia Martinková, Michaela Vařejková, Barbora Miznerová, Markéta Pavlíková, Jana Hlinovská, David Hlinovský, Štěpánka Philippová, Michal Novotný, Karolína Pospíšilová, Paula Biedková, Romana Vojíková, Jan Havlík, Valerie Bríd O'Leary, Marie Černá, Aleš Bartoš, Tom Philipp

**Affiliations:** ^1^Department of Rheumatology and Rehabilitation, Third Faculty of Medicine, Thomayer University Hospital, Charles University, Prague, Czechia; ^2^Department of Statistical Modelling, Institute of Computer Science of the Czech Academy of Sciences, Prague, Czechia; ^3^Department of Neurology, Third Faculty of Medicine, Thomayer University Hospital, Charles University, Prague, Czechia; ^4^Department of Circuit Theory, Faculty of Electrical Engineering, Czech Technical University in Prague, Prague, Czechia; ^5^Department of Physical Medicine and Rehabilitation, Military University Hospital Prague, Prague, Czechia; ^6^Department of Medical Genetics, Third Faculty of Medicine, Charles University, Prague, Czechia; ^7^Department of Neurology, Third Faculty of Medicine, Charles University, Prague, Czechia; ^8^University Hospital Kralovske Vinohrady, Prague, Czechia

**Keywords:** ischemic stroke, lncRNA—long non-coding RNA, motor disability, rehabilitation, physiotherapy, motor recovery, international classification of functioning, disability and health model

## Abstract

**Introduction:**

While the role of physiotherapy as part of a comprehensive inpatient rehabilitation is indisputable, clear evidence concerning the effectiveness of different rehabilitation managements [interdisciplinary implementing the International Classification of Functioning, disability and health (ICF) vs. multidisciplinary model] and physiotherapy categories (neuroproprioceptive “facilitation, inhibition” vs. motor/skill acquisitions using technologies) are still lacking. In this study, four kinds of comprehensive inpatient rehabilitation with different management and content of physical therapy will be compared. Moreover, focus will be placed on the identification of novel biological molecules reflective of effective rehabilitation. Long non-coding RNAs (lncRNAs) are transcripts (>200 bps) of limited coding potential, which have recently been recognized as key factors in neuronal signaling pathways in ischemic stroke and as such, may provide a valuable readout of patient recovery and neuroprotection during therapeutic progression.

**Methods and analysis:**

Adults after the first ischemic stroke in an early sub-acute phase with motor disability will be randomly assigned to one of four groups and undergo a 3 weeks comprehensive inpatient rehabilitation of different types: interdisciplinary team work using ICF model as a guide; multidisciplinary teamwork implementing neuroproprioceptive “facilitation and inhibition” physiotherapy; multidisciplinary teamwork implementing technology-based physiotherapy; and standard multidisciplinary teamwork. Primary (the Goal Attainment Scale, the Patient-Reported Outcomes Measurement Information System, and the World Health Organization Disability Assessment Schedule) and secondary (motor, cognitive, psychological, speech and swallowing functions, functional independence) outcomes will be measured. A blood sample will be obtained upon consent (20 mls; representing pre-rehabilitation molecular) before and after the inpatient program. Primary outcomes will be followed up again 3 and 12 months after the end of the program. The overarching aim of this study is to determine the effectiveness of various rehabilitation managements and physiotherapeutic categories implemented by patients post ischemic stroke *via* analysis of primary, secondary and long non-coding RNA readouts. This clinical trial will offer an innovative approach not previously tested and will provide new complex analysis along with public assessable molecular biological evidence of various rehabilitation methodology for the alleviation of the effects of ischemic stroke.

**Clinical trial registration:**

NCT05323916, https://clinicaltrials.gov/ct2/show/NCT05323916.

## Introduction

Ischemic stroke (IS) is one of the leading causes of neurological dysfunction, the most common being motor disability (1) which negatively impacts life quality and active participation (2, 3).

Undoubtedly, complex rehabilitation (4, 5) is needed. Well-coordinated team work is already taken as a standard approach of complex rehabilitation (6). Multidisciplinary management where specialists work in parallel toward addressing problems related to their profession is frequently the case. An interdisciplinary management where specialists are working as a group to achieve a common goal that is explicitly agreed upon using the International Classification of Functioning, Disability and Health (ICF) model recommended by the World Health Organization (7), is applied rather in Western and Northern European countries, in contrast to other European countries where the use of these tools is rare (8). No study **comparing the effectiveness of an interdisciplinary teamwork implementing the ICF model with multidisciplinary teamwork in stroke** (7, 9) has been carried out so far.

Physiotherapy (PT) as a part of the team rehabilitation work plays an indisputable role in impairment reduction, activity independence, social participation and quality-of-life improvement (5). Many PT techniques have been developed to facilitate the recovery of motor disability in patients after stroke (1, 6, 10, 11). Clear evidence about what kind of PT approaches as part of rehabilitation is more effective is still missing (12). Several studies compared different PT methods (13), but only small beneficial differences between groups were found probably due to a-systematic/accidental indication of PT methods in rehabilitation processes (14, 15). This is a reason why we decided to compare an effectiveness of main PT categories, 1. motor/skill acquisitions using technologies and 2. neuroproprioceptive “facilitation, inhibition” (16). Both are related to plasticity (the capacity of the central nervous system to adapt and change) *via* the development of new neuronal interconnections, acquiring new functions, and compensating for impairment (17). In Motor/skill acquisitions using technologies, the patient is increasingly active in the motor re-training process and the principles of sensory-motor learning are applied. Such approach induces a cortical network reorganization closely related topographically to the trained movement which leads to synaptogenesis (18). In neuroproprioceptive ′facilitation, inhibition′ PT, the effectiveness of the synaptic connections among neurons forming functional networks is enhancing with aim to evoke the movement by a suitable combination of afferent stimuli. It modulates interneuronal systems, repeatedly activates motor programs at the subcortical level, and as such induces adaptive and plastic processes of the CNS (19). Until now, nobody compared an effectiveness of these two PT categories which use different mechanisms to activate processes of the plasticity. The only comparison of technology-based PT with equal intensity of over-ground rehabilitation did not show superiority of one over the other (20).

One of the most used options to investigate adaptive and plastic processes of the CNS are imaging methods as functional magnetic resonance or diffusion tensor imaging. Unfortunately, localization and size of changes following rehabilitation are dynamic and differ between studies (21). Research in other areas suggests that molecular biomarkers could be indicators of improvements in neurobiological principles that support repair or compensatory strategies that stimulate adaptive responses in people after stroke (22). The role of long non-coding RNA (lncRNA), defined as RNA transcripts >200 nucleotides with limited coding potential (23), e.g., *MALAT1, SNHG12, MEG3* and *H19*, in ameliorating IS brain injury has now been recognized (24). *FosDT* might be an important lncRNA for modulation of ischemic neuronal damage, and the inhibition of lncRNA H19 protected cells from OGD/R-induced death by preventing autophagy activation (25). Long non-coding RNA are pivotal factors in neuronal repair processes and enhance neurogenesis. Nevertheless, there is a lack of human studies and clinical trials involving lncRNAs in IS treatment. The medical community has stressed the urgency of implementing studies that may clarify the clinical impact of lncRNAs in the specific context of IS given their promising involvement in nervous system recovery. They have been classified into anti-sense, intronic, large intergenic, promoter associated and UTR-associated lncRNAs (26). They are involved in vital cellular regulation (27) including genomic imprinting (28), epigenetic chromatin modification (29), transcriptional interference (30) and nuclear export (31). Importantly, lncRNAs determine nervous system development (32). A majority of lncRNA display specific expression within neuroanatomical regions (33). Many of these lncRNAs display genomic localizations in close proximity to known neurodevelopmental regulators (34). This has led to the general hypothesis that the expanded diversity in lncRNAs is pivotal to the higher order cognitive ability of humans. LncRNAs regulate key factors involved in ischemic/reperfusion injury, e.g., calcium overload. Excessive calcium accumulation results in the activation of calcium/calmodulin-dependent protein kinase II (CaMKII), a family of serine/threonine kinases involved in IS pathogenesis (35). CaMKII is controlled by lncRNA *C2dat1*. Elevated levels of lncRNA *C2dat1* have been identified in both *in vitro* and *in vivo* models of IS (35). The lncRNA CAMK2D-associated transcript (*C2dat1*) is able to regulate CaMKIIδ, a CaMKII isoform, by targeting CAMK2D-associated transcript (*C2dat1*), thus being able to regulate key factors involved in I/R injury such as calcium overload or glutamate toxicity, and *C2dat1* promoted neuronal survival *via* activation of nuclear factor kappa B (NF-κB) signaling cascade, which may suggest *C2dat1* is a promising therapeutic approach for ischemia. On the other hand, another report showed that the inhibition of CaMKII can prevent 30–70% of ischemia-induced neuronal death; respectively, the regulation of lncRNAs may exert pro-angiogenic, neuroregenerative, anti-apoptotic, and anti-inflammatory effects in injured brain tissue (24). Studies showed that IS injury leads to increased glutamate release activating N-methyl-D-aspartate (NMDA) receptors which initiate cellular apoptosis (36). Overexpression of lncRNA *GAS5* increased the apoptotic rate in neurons with its administration resulting in a greater area of cerebral infarction in animal models (37). It has been suggested that inhibition of the lncRNA *GAS5* could potentially reduce apoptosis and infarct size in IS leading to improved neurological functioning. Evidence has pointed to lncRNA moderation of autophagy, angiogenesis and oxidative stress caused by IS. Exploring lncRNAs involved in such processes may assist in understanding the recovery networks induced by IS rehabilitation approaches.

For association analysis which may provide further evidence identifying indicators of effective rehabilitation, systematic collection of demographics, clinical and molecular marker information is important. Until now, there is limited evidence to guide judgements regarding the rehabilitation potential following stroke (24, 38–41). An intensity of rehabilitation program appears to be one the most suitable predictor of its effectivity (40, 42), but it is unclear if high intensity programs such as the intensive comprehensive program with a minimum duration of 4 h a day recommended by the Czech Ministry of Health (43) is beneficial or all patients. However, there is limited evidence, whether such intensive program is more effective than the comprehensive rehabilitation program standardly offered. Research also shows that some clinical features such as the level of consciousness, severity of hemiplegia, incontinence, dysphagia and dysphasia (44) or disease severity (45, 46) may be considered as indicators as to whether the rehabilitation process could be effective, however, there exists a certain amount of confusion between predicting natural unassisted recovery and predicting responsiveness to targeted rehabilitation (38).

The current clinical trial aims at filling the above-mentioned research gaps in effectiveness of rehabilitation after stroke. The primary aim is to compare the effectiveness of an interdisciplinary teamwork implementing the ICF model with multidisciplinary teamwork in stroke. As the next aim, we are interested whether PT using principles of sensorimotor learning implementing technology is more effective than neuroproprioceptive “facilitation, inhibition” PT. We focus on PT categories instead of on specific intervention methods, however, information about specific intervention (e.g., Bobath concept, Vojta reflex locomotion) each patient underwent will be recorded and included in the analysis. As the third aim, we focus on the identification of novel biologicals reflective of effective rehabilitation by molecular assessment, in order to answer the question whether the long non-coding RNA is a suitable biomarker for documenting the plastic and adaptive processes of the CNS. Finally, using a uniquely complex dataset, we aim to research what factors play role in effective post stroke rehabilitation and which clinical tests and scales are the most useful in documenting effectiveness of the rehabilitation, even from a long-term perspective.

This clinical trial will test the following scientific hypotheses:

I. COMIRESTROKE under all four settings has a positive influence on all measured outcomes, both in short- and in the long-term. Intensive complex rehabilitation (COMIRESTROKE ICF, NEFI, TECH) has higher effect on all outcomes than standard complex rehabilitation (COMIRESTROKE control).
II. Interdisciplinary management implementing ICF model (COMIRESTROKE ICF) has higher effect than multidisciplinary management (COMIRESTROKE NEFI. TECH, CONTROL) on primary outcomes (GAS, PROMIS, WHODAS 2.0) and on such secondary outcomes that were identified as treatment goals for individual patient. Furthermore, we expect the highest impact on the primary outcomes in the follow-up (three and 12 months after finishing rehabilitation).
III. COMIRESTROKE—NEFI has higher effect than COMIRESTROKE—TECH on the secondary outcomes, mainly on motor functions. Moreover, it leads to the stronger initiation of plastic and adaptive processes, as assessed by the level of lncRNAs in the peripheral blood.
IV. Changes in molecular biological readouts will correlate with changes in clinical parameters and will be the most sensitive to document effect of the therapy.
V. The most important predictor of effective rehabilitation is the level of disability at admission time. However, the category of the rehabilitation has an impact on perceived, clinical, and physiological changes of the rehabilitant.

Moreover, we have these two additional exploratory goals:

Goal I—Improvement patterns: Considering the high number of measured outcomes, for a deeper understanding of therapy efficacy with respect to patient and treatment characteristics, this clinical trial will aim to identify groups of patients with similar improvement patterns post therapy.
Goal II—Item-level analysis: To provide a deeper understanding of the differences in effectiveness between the three therapeutic approaches, this clinical trial aims to explore item-level between-group differences in improvement.

Research from other areas also suggests that between-group differences in improvement may be found on an item level of multi-item measurements even in cases when they are not observed on the total scores, and thus, a more detailed item-level analysis may provide an important insight.

## Methods and analysis

### Study design

**Figure 1:**
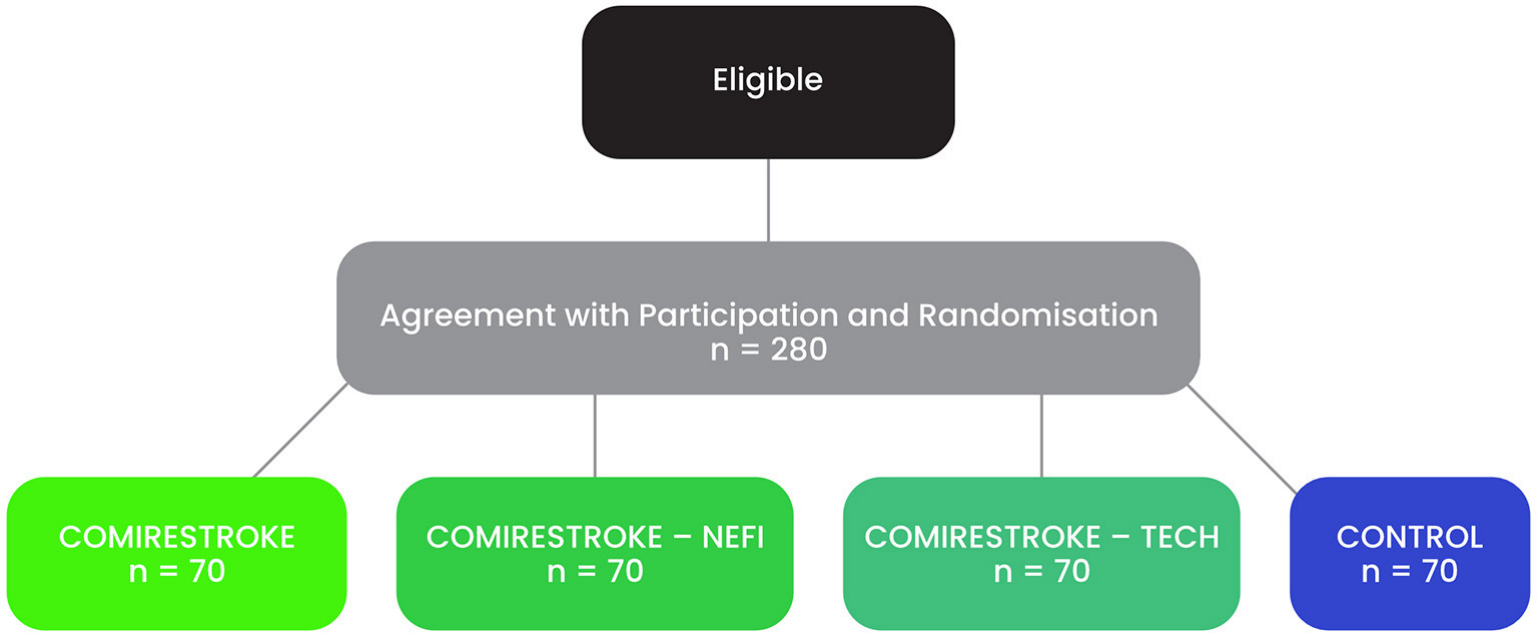
Study design.

This will be a Four-Arm Parallel-Group Randomized Double Blinded Controlled Trial with a longitudinal design. Patients who fulfilled inclusion criteria will be randomly assigned to one of four research groups (Group 1, 2, 3, or 4). Participants will be examined four times (Figure 1). Primary and secondary outcomes will be measured, and a blood sample will be obtained before and after the inpatient program. Primary outcomes and a blood sample will be followed up again 3 and 12 months after the end of the program (Table 1).

**Table 1:** Study design.

	**STUDY PERIOD**					
	**Enrolment**	**Allocation**	**Post-allocation**			
TIMEPOINT**	**0**	**0**	***t_1_***	***t_2_***	***t_3_***	***t_4_***
			***Day***	***Day***	***Month 3***	***Month 12***
			***1–2***	***19–20***		
**ENROLMENT**						
Eligibility screen	X					
Informed consent	X					
Allocation		X				
**INTERVENTIONS**						
COMIRESTROKE—ICF			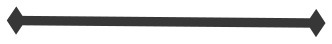			
COMIRESTROKE—NEFI			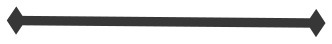			
COMIRESTROKE—TECH			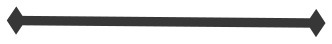			
COMIRESTROKE—CONTROL			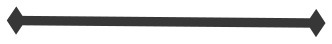			
**ASSESSMENTS**						
Basic characteristics	X					
Primary outcomes			X	X	X	X
Secondary outcomes			X	X		
Molecular biological readouts			**X**	**X**	**X**	**X**

COMIRESTROKE: COMprehensive Intensive REhabilitation Programs after STROKE; COMIRESTROKE—ICF: COMIRESTROKE using the International Classification of Functioning, disability and health model as a guide; COMIRESTROKE—NEFI: COMIRESTROKE implementing neuroproprioceptive “facilitation and inhibition” physiotherapy; COMIRESTROKE—TECH: COMIRESTROKE implementing technology-based physiotherapy; CONTROL—standard inpatient care.

The SPIRIT reporting guidelines were used when developing the protocol (47). Moreover, recommendation of the Czech Ministry of Health (43) influenced the protocol in (1) the definition of the rehabilitation intensity in interventions cohorts COMIRESTROKE ICF + NEFI + TECH (minimally 4 h of rehabilitation per day), (2) the choice of methods for comprehensive clinical testing, and (3) implementation of technology-based PT. Our department wanted to improve clinical care about people after stroke and decided to implement this best practice. While preparing a clinical project based on this document (43), our scientifically unresolved questions, that we presented in the introduction, we encountered.

### Participants

Participants will be identified by a neurologist based on *inclusion criteria*: Adults (18–85 years) after the first IS in early sub-acute phase, specifically 7 days-3 months (48, 49)—phase the most important for recovery involving spontaneous improvement as well as benefits arising from previous therapeutic intervention (11), with a slight to moderately severe disability {2–4 on the Modified Rankin Scale (50)}, with minimal or moderate motor deficit of upper or lower extremities (on NIHSS Item 5 or 6 scores 1–3 points) who were able to perform activities of daily living prior to stroke event {0–2 on the Pre-Stroke Modified Rankin Score (51)}, with a potential to accept 4 h of comprehensive rehabilitation per day and to profit from the physiotherapy. Czech is their native language or its knowledge is at the level of the mother tongue.

#### Exclusion criteria

Low level of consciousness (vegetative state and/or minimally conscious state); severe cognitive decline that would interfere with administration of the tests, premorbid illiteracy, severe visual and/or auditory deficit that would prevent proper completion of the tests; behavioral disorders and/or lack of cooperation with therapist; and severe medical problems with a poor prognosis (e.g., severe frailty, advanced and incurable cancer, fracture, cardiovascular disorders as chronic heart failure NYHA III, IV, symptomatic coronary artery disease, angina severity class III, IV, respiratory insufficiency as chronic obstructive pulmonary disease GOLD IV, and another severe disease) (52).

#### Randomization

All patients meeting the above criteria will be invited to participate and asked to provide a written informed consent. They will be randomly assigned (1:1:1:1) as soon as possible, but always within 48 h of admission, into one of the four interventions (represented by Group 1, 2, 3 or 4) using offsite-independent randomization protocols (www.randomization.com). Concealed allocation will be performed using sequentially numbered opaque sealed envelopes only accessible by research personnel with no involvement in the trial.

#### Estimated size

**Figure 2:**
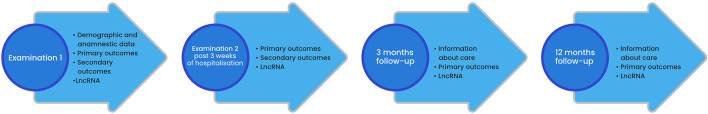
Planned Recruitment and Randomization Process.

It is estimated that ~140 people will be recruited to the clinical trial each year. This number is based on the fulfillment of performance and quality indicators report of cerebrovascular care at the Center for Highly Specialized Patient Care for Stroke Patients at Thomayer Hospital 2019. A total of 280 people is expected to be enrolled in the clinical trial over a period of 2 years (Figure 2), corresponding to a sample number of 70 patients per group. This is sufficient to detect a difference between two groups with the effect size of 0.5 (Cohen's d, considered as moderate to large effect), a significance level of α = 0.05 and a strength of 1-β = 0.80. To detect the effect of therapy within group, required sample size would be even lower as paired *t*-test will be used. Considering an effect size of 0.5 for WHODAS 2.0 scores is appropriate according to previous studies (53).

### Interventions

All participants will undergo a complex 3 weeks inpatient rehabilitation. Group 1, 2, 3 undergo intensive rehabilitation (4 h/day for 3 weeks) while intensity in group 4 will be defined by medical doctor (standard procedure). Management of Group 1 will be interdisciplinary, implementing ICF model, while in Group 2, 3 and 4 will be multidisciplinary managed. PT in group 2 will be based on principles of neuroproprioceptive “facilitation inhibition” and in Group 3 based on sensorimotor learning (motor/skill acquisition using technologies) while content of group 4 will not be pre-defined, but it will be indicated by the medical doctor (Table 2).

**Table 2:** Main differences in the intervention between four groups.

	**Rehabilitation management**	**Intensity of rehabilitation**	**Content of PT**	**Length of PT**
Group 1 COMIRESTROKE—ICF	Interdisciplinary implementing ICF model	4 h per day	Individually given on rehabilitation conference according to patients wishes	Adapted to the set goal
Group 2 COMIRESTROKE—TECH	Multidisciplinary	4 h per day	Implementing technology-based PT	1 h
Group 3 COMIRESTROKE—NEFI	Multidisciplinary	4 h per day	Implementing principles of neuroproprioceptive “facilitation, inhibition”	1 h
Group 4 COMIRESTROKE—CONTROL	Multidisciplinary	Adapted by medical doctor	Adapted by medical doctor	Adapted by medical doctor

Therapists in each group will be maximally helpful and will adopt a schedule for each patient to complete all the sessions. The treatment in each session will be led in person by well-educated, experienced therapists especially trained in each method. Rehabilitation team working with cohort 1 underwent 2 days on-line worskhop (54) and work under supervision of Czech specialist in the ICF concept (55).

The treatment will be modified according to the patient's status and reaction to the therapy. Physical load during all therapies will be perceived maximally as a moderate level of intensity {12 on the Borg Scale (56)}. Information about the treatment (frequency, length and content of each treatment session) will be recorded using codes of Public Health Insurance, Czech Republic (codes correspond to The Current Procedural Terminology code set system) (Table 3). Moreover, content of PT will be defined using Vocabulary of PT interventions (16).

**Table 3:** Information about the treatment.

**CODE**	**NAME**	**DURATION**
**REHABILITATION DOCTOR**		
21021	COMPREHENSIVE EXAMINATION BY A REHABILITATION DOCTOR	60
21022	TARGETED EXAMINATION BY A REHABILITATION DOCTOR	30
21023	CONTROL EXAMINATION BY A REHABILITATION DOCTOR	15
**PHYSICAL THERAPIST**		
21001	COMPLEX KINESIOLOGICAL EXAMINATION	45
21002	KINESIOLOGICAL EXAMINATION	30
21003	CONTROL KINESIOLOGICAL EXAMINATION	20
21020	THERAPY ON DEVICES USING THE PRINCIPLE OF BIOLOGICAL FEEDBACK	15
21030	KINESIOTHERAPY USING ROBOTIC TECHNOLOGY FOR VERTICALIZATION AND WALKING TRAINING	30
21032	KINESIOTHERAPY USING ROBOTIC TECHNOLOGY—UPPER LIMBS THERAPY	30
21116	LOCAL INSTRUMENT CRYOTHERAPY	20
21211	THERAPEUTIC PHYSICAL EDUCATION GROUP TYPE I., 3–5 TREATED	15
21213	THERAPEUTIC PHYSICAL EDUCATION GROUP TYPE II., 6–12 TREATED	15
21215	THERAPEUTIC PHYSICAL EDUCATION—INSTRUCTION AND TRAINING OF THE PATIENT AND HIS FAMILY MEMBERS	30
21217	THERAPEUTIC PHYSICAL EDUCATION GROUP IN A POOL WITH HOT WATER-−15 MIN	15
21219	THERAPEUTIC PHYSICAL EDUCATION INDIVIDUAL UNDER SUPERVISION OF INSTRUMENTS	15
21221	INDIVIDUAL KINESIOTHERAPY I.	45
21225	INDIVIDUAL KINESIOTHERAPY II.	15
21413	SOFT TISSUE TECHNIQUES	15
21415	MOBILIZATION OF THE SPINE AND PERIPHERAL JOINTS	15
21510	SOFT AND MOBILIZATION TECHNIQUES	15
21520	MOBILIZATION OF THE SPINE OR JOINT—WITH AN IMPACT	10
21530	DETERMINATION OF THE LONG-TERM REHABILITATION PLAN BASED ON THE PAST REHABILITATION CONFERENCE	90
21711	BACK SCHOOL—PREVENTION OF RECURRENCE OF VERTEBROGENIC DISEASES	90
21713	REFLECTIVE AND LIGHTER MASSAGE	30
21715	REEDUCTION OF MOVEMENT DIAGRAMS AND HABITS AND THEIR CORRECTIONS	20
21717	INDIVIDUAL LOCOMOTION AND MOBILITY TRAINING	15
**OCCUPATIONALTHERAPIST**		
21611	ERGOTHERAPEUT EXAMINATION AT THE BEGINNING OF ERGOTHERAPY	45
21612	CLASSIFICATION ACCORDING TO THE INTERNATIONAL CLASSIFICATION OF FUNCTIONALITIES, DISABILITY AND HEALTH	30
21613	ERGOTHERAPEUT CONTROL EXAMINATION	30
21614	ERGOTHERAPEUTIC EXAMINATION FOR DESIGN OF SUITABLE TECHNOLOGIES AND PRODUCTS	20
21621	INDIVIDUAL BASIC ERGOTHERAPY	30
21622	ERGOTHERAPEUTIC METHODS ON A NEUROPHYSIOLOGICAL BASIS	45
21623	INDIVIDUAL ERGOTHERAPY USING WORKSHOPS	30
21625	TRAINING OF EVERYDAY DAILY ACTIVITIES—ADL	30
21627	BASIC ERGOTHERAPY BASIC	11
21629	GROUP ERGOTHERAPY USING WORKSHOPS	11
21631	TARGETED ERGOTHERAPY OF THE HAND	30
**SPEECH THERAPIST**		
72015	COMPREHENSIVE EXAMINATION BY A CLINICAL SPEECH THERAPIST	40
72016	TARGETED EXAMINATION BY A CLINICAL SPEECH THERAPIST	30
72017	CONTROL EXAMINATION BY A CLINICAL SPEECH THERAPIST	15
72019	SPEECH THERAPY SUPPLEMENTARY COMPREHENSIVE AND CONTROL EXAMINATIONS	40
72211	SPEECH THERAPY PROVIDED BY A SPEECH THERAPIST IN AN OUTPATIENT EQUIPMENT OR IN A STATIONARY	30
72213	SPEECH THERAPY SPECIALLY DEMANDING PROVIDED IN HOSPITALIZATION	45
72215	SPEECH THERAPY MEDIUM DEMANDING PROVIDED IN HOSPITALIZATION	45
**PSYCHOLOGICST**		
37021	COMPLEX PSYCHOLOGICAL EXAMINATION	60
37022	TARGETED PSYCHOLOGICAL EXAMINATION	60
37023	CONTROL PSYCHOLOGICAL EXAMINATION	30
37111	SPECIFIC PSYCHOLOGICAL INTERVENTION	30
37121	PSYCHODIAGNOSIS WITH DEMANDING PSYCHOTHERAPEUTIC INTERVENTION	90

#### Group 1: COMIRESTROKE—ICF (interdisciplinary team management implementing ICF system)

Participants will undergo intensive (4 h per day) rehabilitation where the interdisciplinary team of professionals from different clinical fields (physiotherapy, occupational therapy, clinical speech therapy and psychology) will co-operate using the ICF management tools (such as the ICF Intervention Table, defining and modifying therapeutic interventions in respect to the paitent's goals and sub-goals and in concordance with the functional profile agreed on rehabilitation conferences). These ICF tools are used for systematic organization of the interdisciplinary team.

First therapeutic day, a functional profile will be created by interdisciplinary team and rehabilitator on rehabilitation conference to take into account the functioning, activity and participation level of the patient (57–59).

In order to limit the burden of repetitive clinical examinations, questionnaires surveys and completion of documentation, this study proposes direct linking of some normal-range clinical test results to the codes included in the Comprehensive ICF Stroke Brief set (60) and Rehabilitation set (ICF 30 generic set) (61) (Table 4).

**Table 4:** An example of linking of clinical test results to the codes.

**ICF category**	**Title**	**BF-qualifier**	**Source: Case history**	**Source: Patient reported questionnaire**	**Source: Clinical examination**
b110	Consciousness functions	8			NIHSS, mRS, MoCA
b114	Orientation functions	8			MAST, MoCA, ALBA, PICNIR
b117	Intellectual functions	8			MAST, MoCA, GUSS
b140	Attention functions	8		WHODAS	NHPT, ARAT, GUSS, MAST, MoCA, ALBA, PICNIR
b144	Memory functions	8		WHODAS, FIM	MAST, MoCA, ALBA, PICNIR
b156	Perceptual functions	8			NHPT, ARAT, GUSS
b164	Higher-level cognitive functions	8			MoCA, MAST, GUSS, ALBA, PICNIR
b167	Mental functions of language	8			MAST, MoCA, ALBA, PICNIR
b172	Calculation functions	8			MoCA
b280	Sensation of pain (G)	8		WHODAS	
b310	Voice functions	8			3F
b320	Articulation functions	8			3F, MAST, mRS
b330	Fluency and rhythm of speech functions	8			3F, MAST
b455	Exercise tolerance functions	8			6 m
b510	Ingestion functions	8			GUSS, mRS
b525	Defecation functions	8		FIM	
b620	Urination functions	8		FIM	
b640	Sexual functions	8		WHODAS	
b750	Motor reflex functions	8			mRS
b755	Involuntary movement reaction functions	8			TUG, BBS, mRS
b760	Control of voluntary movement functions	8		MAL	Nine Hole Peg Test, ARAT, TUG, BBS, 10 m, 6 m, mRS
b770	Gait pattern functions	8			TUG, BBS, 10 m, 6 m
s750	Structure of lower extremity				TUG, BBS, 10 m, 6 m
d115	Listening			WHODAS	

The codes included in the Comprehensive ICF Stroke Brief set (marked in Green) and Rehabilitation set (ICF 30 generic set) (marked in beige), an example on first thirteen ICF categories.

The interdisciplinary team together with the patient will use the functional profile to define in detail the overall goal and sub-goals of the treatment (62, 63), and to determine specific therapeutic interventions to achieve them (42).

The whole team will work on defined goals, so the time spent with each specialist will depend on the individually set goals. An “ICF Intervention Table” (64) will be used to monitor and evaluate the work during individual targeted interventions and will also serve as a means of communication between the members of the interdisciplinary team. Length of PT will be adapted to the set goal. If the patient wishes to improve some motor skill, it will be longer than if he wishes to improve, for example, speech.

The team will meet weekly (every Monday) on rehabilitation conference to provide feedback, evaluate the fulfillment of the set goals and to adjust therapeutic procedures so that the goals of the therapy are best met.

After finishing hospitalization, participants will undergo a 1-month outpatient rehabilitation programme based on the ICF concept and later will receive standard health and social care.

#### Group 2: COMIRESTROKE—TECH (motor/skill acquisition implementing technology-based PT)

Participants will undergo intensive (4 h per day) rehabilitation where the multidisciplinary team of practitioners from different clinical fields (physiotherapy, occupational therapy, clinical speech therapy and psychology) will be led by a medical doctor, a specialist in rehabilitation and physical medicine.

One therapeutic intervention (60 min) will be pre-defined as individual PT using principles of sensorimotor learning, i.e., repeated specific and targeted functions in different environments/conditions in order to learn trained motor function, strengthen memory footprint for this function, and as such to initiate plastic and adaptive processes in the CNS. According to the motor function indicated by PT to learn (for example walking or drinking), the most appropriate technology will be chosen, for example robotic systems using an exoskeleton (Gloreha, Erigo and Meditutor) or a therapy applied in virtual environment (65, 66).

After finishing hospitalization, participants will undergo standard/not directed treatment offered by the Czech Health Care system.

#### Group 3: COMIRESTROKE—NEFI (PT implementing neuroproprioceptive “facilitation, inhibition”)

Participants will undergo intensive (4 h per day) rehabilitation where the multidisciplinary team of practitioners from different clinical fields (physiotherapy, occupational therapy, clinical speech therapy and psychology) will be led by a medical doctor, a specialist in rehabilitation and physical medicine.

One therapeutic intervention (60 min) will be pre-defined as individual PT based on principles of neuroproprioceptive “facilitation, inhibition” PT, it means methods like Vojta reflex locomotion (67), Bobath concept (68), Proprioceptive Neuromuscular Facilitation (61), Motor Program Activating Therapy (69), etc. All these methods apply the appropriate stimuli in different postural positions in order to activate optimal motor function and ability control. They modulate neuronal threshold, and as such induces adaptive and plastic processes of the CNS.

After finishing hospitalization, participants will undergo standard/not directed treatment offered by the Czech Health Care system.

#### Group 4: COMIRESTROKE—CONTROL

Participants will undergo standard multidisciplinary care including face to face physiotherapy (bed mobility, transfers, gait, therapeutic exercises, positioning, education) recommended by medical doctor.

After finishing hospitalization, participants will undergo standard/not directed treatment offered by the Czech Health Care system.

### Pre- and post-intervention assessments

Once an informed consent is obtained prior to randomization; participants will be referred to study examiners—a physical medicine and rehabilitation physician, a neurologist, a physical therapist, a speech therapist, an occupational therapist and a psychologist, who will not know the treatment group of the patient. They will administer baseline testing during the 2nd and 3rd day after admission to the department (Pre-assessment). Post-assessment 1 will be done at the end of the 3-week inpatient intensive comprehensive rehabilitation (during the last 2 days of hospitalization). Follow-up assessments will take place 3 and 12 months after admission, respectively, by a telephone interview or hospital visit. The aim is for each of the measurements to be assessed by a single examiner only (Table 5); in case of more examiners, inter-rater reliability will be assessed.

**Table 5:** Examination timing and distribution by the team.

**Examination**	**Abbreviation**	**Examination Number**			
		**1**	**2**	**3**	**4**
		**Baseline**	**+3 weeks**	**+3 months**	**+ 1 year**
**Neurologist**					
Inclusion and exclusion criteria form		X			
Patients' characteristic					
Modified rankin scale	mRS	X	X		
Pre-stroke Modified Rankin Score	pre-stroke mRS	X			
National Institute of Health Stroke Scale	NIHSS	X	X		
Psychologist*					
Montreal Cognitive Assessment	MoCA	X	X		
Amnesia Light and Brief Assessment	ALBA	X	X		
NEO-Five Factor Inventory	NEO - FFI	X			
Patient-reported Outcomes Measurement Information System Global Health**	PROMIS	X	X	X	X
The World Health Organization Disability Assessment Schedule **	WHODAS	X	X	X	X
The Goal Attainment scale	GAS	X	X	X	X
Picture Naming and Immediate Recall	PICNIR	X	X		
Neuro—QOL depression	DEP	X	X		
**Physiotherapist**					
Hand Dynamometer	Strength	X	X		
Postural tremor	Tremor	X	X		
Action Research Arm Test*	ARAT	X	X		
Motor Activity Log*	MAL	X	X		
Timed Up and Go*	TUG	X	X		
Berg Balance Scale*	BBS	X	X		
The 10 Meter Walk Test*	10 m	X	X		
The 6 min Walk Test*	6 min	X	X		
Functional Independence Measure*	FIM	X	X		
Laterality index	LI	X			
Nine Hole Peg test	NHPT	X	X		
Speech therapist*					
The Gugging Swallowing Screen	GSS	X	X		
The 3F Test – Dysarthric Profile	3F test	X	X		
Dysarthria Analyzer	Dysan	X	X		
The Mississippi Aphasia Screening Test	MAST	X	X		
Image Naming Test	INT	X	X		
**Long Non-coding RNA assessment**	*C2dat1*	X	X	X	X
Blood sample isolation	*GAS5*	X	X	X	X
Total RNA extraction	*MALAT1*	X	X	X	X
cDNA synthesis	*PARTICLE*	X	X	X	X
LncRNA and endogenous gene QPCR	*H19*	X	X	X	X
LncRNA expression analysis (2^−ΔΔCt^)	*MEG3*	X	X	X	X

^*^Tests recommended by Registration sheet of the treatment day following comprehensive intensive rehabilitation treatment for patients with acquired brain damage (43). ^**^Primary outcomes.

A wide range of patient characteristics will be collected to address all functions and activities of the patient. To provide systematic categorization (70), the ICF model (6) has been chosen. However, many different measures are used to address different levels of the ICF. Therefore, a team of experienced clinicians and researchers participated in the selection of the most suitable tests into the methodology. Choosing was influenced by psychometric properties, appropriateness of the instrument, but also by use of test in specific regional contexts (71).

We considered the patients' subjective feelings about how they have improved to be the most important aspect. Therefore, the Goal Attainment scale (GAS) together with the Patient-Reported Outcomes Measurement Information System (PROMIS) Global Health, and the World Health Organization Disability Assessment Schedule (WHODAS 2.0) have been chosen as primary outcomes. As secondary outcomes, motor, cognitive, psychological, speech and swallowing functions as well as functional independence will be measured. When choosing secondary outcomes, we based on recommendation of Czech Ministry of Health (43). In this document, list of recommended tests for physical therapists and occupational therapists was presented. Specifically, Action Research Arm Test, Motor Activity Log, Timed Up and Go, Berg Balance Scale, The 10 Meter Walk Test, The 6 Min Walk Test and Functional Independence Measure. They have been supplemented by objective examinations like Nine Hole Peg Test, the Hand Dynamometer and Accelerometer (72) which may provide more extensive information. Assessment of clinical psychologists and speech therapists was not defined in this recommendation and was chosen by expert consensus.

**Demographic and anamnestic data** including personal factors (sex, age, education, occupation, social relations, living situation), weight and height, laterality index (73), number of days after the stroke event, personality based on the NEO-Five Factor Inventory (NEO-FFI) (74), pre-stroke functional status based on modified Rankin Scale (75), cardiovascular risk factors (76), the degree of neurological impairment according to the National Institute of Health Stroke Scale (77) and disability with the Modified Rankin Scale (50), Bamford classification of IS based on the initial presenting symptoms and clinical signs (78) as well as pharmacotherapy will be collected.

### Primary outcomes

As primary outcomes, scales documenting the patients' subjective feelings about how they have improved will be be chosen.

- PROMIS Global Health is a 10-item scale which asks the patient to assess (self-report) their physical, mental and social health in the past 7 days (79, 80).
- WHODAS 2.0 is a generic assessment instrument developed by WHO to provide a standardized method for measuring health and disability. It is grounded in the conceptual framework of the ICF and integrates an individual's level of functioning in major life domains and directly corresponds with ICF's “activity and participation” dimensions. The 36-item version will be used. A higher score means greater disability (81).
- GAS is an individualized outcome measure involving goal selection and goal scaling that is standardized in order to calculate the extent to which a patient's goals are met. Each goal is rated on a 5-point scale (-2 much less than expected, 0 achieved the expected level, 2 much more than expected (82, 83).

Three types of goals will be established:

A. Overarching long-term goal (Global Goal, G) that reflects a desired improvement on the level of participation: usually restoration of previous life including remunerative employment, sport and leisure activities (interview by call at 3 and 12 months follow up).
B. Mid-term goal (Program Goal, P) that reflects improvement mainly in the domain of activities and participations achievable by the rehabilitation program: restoration of self-care (almost), independence in daily living, etc. (interview by call at 3 months follow up).
C. Three Short-term goals (Cycle Goals C1, C2, C3) mainly in the domain of functioning and activities. Usually specific, most problematic components of the Program Goal (evaluated by the rehabilitation team together with the rehabilitant).

### The secondary outcomes

The secondary outcomes include clinical tests and questionnaires of physical functions and functional independence (examined by a physical therapist), speech and swallowing (examined by a speech therapist), cognitive and psychological functions (examined by a clinical psychologist).

#### Upper extremity functions

Jamar Hydraulic Hand Dynamometer will be used to measure isometric grip force from 0 to 90 kg. Five handle positions from 35 to 87 mm will be tested. The measurement is in kg (the higher the value, the better the function) (84).

A postural tremor will be measured by the 3-axis accelerometer and 3-axis gyroscope chip (Motion Tracking sensor MPU-6050) which can measure acceleration up to 16 g and rotation up to 2,000 degrees per second. The sensor will be fixed to the patient using a ring on a finger during stretching the whole arm forward, separately passed for the left and right hand and with opened and closed eyes (1-min measurement for each position). Data from the chip will be acquired by an own measuring device with microcontroller Atmel Mega 328 and stored on an SD card.

For the signal analysis, the magnitude of acceleration—the root of the sum of each component squared—will be computed from separate axes. The sampling frequency will be 100 Hz. Thus, four signals with 6,000 samples will be recorded for each patient—records of postural tremor for right/left hand with opened/closed eyes.

For each record, the signal of acceleration will be filtered by a filter of isoline (typically by high-pass 2nd order Butterworth filter with cut-off frequency of 0.5 Hz). Consequently, the power spectral density (PSD) will be estimated.

The spectral characteristic will be parameterized by selected parameters, for example fMAX (a frequency for which the smoothed PSD is maximal—lower value, lower tremor) or Pf1–f2 (a power of the signal in band from f1 to f2—lower value, lower tremor) (72).

Nine Hole Peg Test is used to measure finger dexterity. A client takes the pegs from a container, one by one, and places them into the holes on the board, as quickly as possible. Shorter times reflect better functioning (85).

Action Research Arm Test (ARAT) is a 19 item observational measure to assess upper extremity performance (coordination, dexterity and functioning). Items are categorized into four subscales (grasp, grip, pinch and gross movement). A higher score means better functioning (86).

Motor Activity Log is a scripted, structured interview to measure real-world upper extremity function consisted of 14 activities of daily living such as using a towel, brushing teeth, and picking up a glass. A higher score means better functioning (87).

#### Mobility and walking

Timed Up and Go is a simple performance-based measure of dynamic balance. The subject stands up from a chair, walks 3 meters, turns back, and sits down again as quickly and safely as possible while being timed. Shorter times reflect better mobility (88).

Berg Balance Scale is a 14-task scale that requires subjects to maintain their balance in positions and tasks of increasing difficulty. A lower score means lower balance capability (89).

The 10 Meter Walk Test is a performance measure used to assess walking speed in meters per second over 10 meters. Shorter time reflects better mobility (90).

The 6 Min Walk Test is a long walking capacity test recording the maximal distance a subject can walk at the fastest speed possible in 6 min. The more distance covered, the better the walking performance (90).

#### Functional independence

Functional Independence Measure is an 18-item measurement tool which explores an individual's physical, psychological and social function. It uses the level of assistance an individual needs to grade functional status from total independence to total assistance. The higher the score, the greater independence capacity (91).

#### Speech and swallowing

The Gugging Swallowing Screen is a simple stepwise bedside screen that allows a graded rating with separate evaluations for non-fluid and fluid nutrition starting with non-fluid textures. It assesses the severity of aspiration risk. A higher score means better functioning (91).

The 3F Test—Dysarthric Profile consists of three subtests (Faciokinesis, Phonorespiration, Phonetics). The overall Index of Dysarthria (ID) is a sum of 45 items with the maximum score of 90. A higher score means better functioning (92).

Following previously published guidelines (93), the dysarthria assessment is based on the automatic evaluation of utterances, including sustained phonation, speech diadochokinetic task, and connected speech. Utterances will be recorded during the sessions with speech language pathologist. The recording will take place in a quiet room with low ambient noise using a head-mounted condenser microphone (Shure Beta 53, Niles, Illinois, U.S.), placed ~5 cm from the mouth corner at an angle of 70°. The recordings will be sampled at 48 kHz with 16-bit resolution. The automatic analysis will be performed using the beta version of the freely available Dysarthria Analyzer (Czech Technical University in Prague, available at http://dysan.cz/).

The Mississippi Aphasia Screening Test (MAST) was developed as a brief, repeatable screening measure for individuals with severely impaired communication/language skills. It has nine subtests that range from 1 to 10 items per subscale. The Index scores sum to 50 points each and are added for the MAST Total Score (0–100 points). The higher the score, the better functioning (94).

Image Naming Test (Test pojmenování obrázku, TPO) is a test of confrontational naming of nouns and verbs. Words are selected based on success, frequency of occurrence, age of adoption, length, and visual complexity. The maximum sum is 60 points (30 verbs and 30 nouns), the results can be assessed qualitatively according to the type of unexplained words (95).

#### Cognitive and psychological functions

The Montreal Cognitive Assessment (MoCA) is an assessment for detecting cognitive impairment ranging from 0 and 30 points. A higher score equates to developed cognition. The Czech version will be validated with cut-offs and norms established (96, 97).

The Amnesia Light and Brief Assessment (ALBA) is an original Czech and innovative test to assess more cognitive functions including short-term memory recall during 3 min. The ALBA consists of four tasks: (1) repetition and encoding of a six-word sentence “Indian Summer Brings First Morning Frost”, (2) sequential demonstration of six gestures, (3) their immediate recall, and (4) final recall of the original sentence. The first task of a sentence repetition reflects (1) language (impaired in aphasia) or (2) encoding and working memory (impaired in memory and attention deficits). The second task of gesturing can be impaired as a result of sensory aphasia or apraxia. Short-term and episodic memory is measured in two different ways, (1) in the third part of the ALBA, i.e., incidental memory of the gestures, and (2) in the fourth part, i.e., intentional verbal memory of the sentence. Overall memory can be expressed as the ALBA memory score, which is a sum of correctly recalled sentence words and gestures. The higher scores of each ALBA part reflect better cognitive functioning. Scores of individual parts range from 0 (the worst) to 6 points (the best) for each of four tasks: (1) the number of correctly repeated words of the sentence (Word 1 score: 0–6 points), (2) the number of correctly recalled words of the sentence after the distraction using the TEGEST (W2 score: 0–6), (3) the number of correctly performed gestures of the TEGEST (Gesture 1 score: 0–6), and (4) the number of correctly recalled gestures of the TEGEST (G2 score: 0–6). The sum, called ALBA memory score, is derived from correctly recalled words of the sentence and correctly recalled gestures together [W2 + G2: 0–12 (6 + 6)]. Example scores of the ALBA test can be the following: 5/1 + 5/3 (W1/2 + G1/2) that gives a total ALBA memory score: 4 (1 + 3) points (98, 99). The ALBA educational video is freely available at: https://www.youtube.com/watch?v=LyCuWc0-Gro.

PICture Naming and Immediate Recall (PICNIR) is an original Czech test certified by the Ministry of Health of the Czech Republic in 2017. The purpose of the PICNIR is to evaluate written speech, long-term sematic and short-term memory simultaneously and quickly up to 5 min. The test consists of two parts. The task of an examinee is to write down names of 20 black and white pictures in one word and remember them at the same time. Then they are asked to rewrite as many picture names as they can recall during 1 min. The results of the PICNIR include a number of wrongly named or unnamed pictures in the first naming part and a number of correctly recalled picture names in the second recall part. The less named errors and more recalled picture names indicate higher cognitive functioning (100). The PICNIR educational video is freely available at: https://www.youtube.com/watch?v=cbJGtPG-nVA.

Neuro–QOL depression is a self-report of health-related quality of life (101).

### Other pre-specified outcome measures—molecular biological readouts of rehabilitation

Blood will be taken for total RNA extraction, cDNA synthesis and real-time QPCR assessment of lncRNA candidates previously identified as potential therapeutic influencers in IS. Whole blood will be collected from fasting patients for RNA analysis. A RiboPure™-Blood Kit (cat# AM1928, ThermoFisher Scientific) will be used for isolating high-quality RNA directly from whole blood. This kit contains an RNA*later*^®^ Solution (cat.# AM7020, ThermoFisher Scientific) that protects RNA and is designed to eliminate the need to process samples as soon as they are harvested. RNA*later*^®^ Solution also “freezes” the gene expression profile of the cells. Treated samples can be safely stored at ambient temperature for extended periods of time (up to 3 days or more). Blood samples stored in RNA*later*^®^ Solution yield RNA of comparable quality to blood samples processed directly according to the commercial website. Expected average yields of total RNA will be between 2 and 4 μg/0.5 mL of whole blood. Total RNA will be reverse transcribed into cDNA. Human lncRNA and internal endogenous gene (e.g., *GAPDH*) expression will be quantified using RNA extracted from blinded samples (i.e., concealment of group allocation) to eliminate bias.

### Statistical analysis

#### Descriptive statistics

On the baseline, the distribution of all primary and secondary outcomes will be visualized using histograms and QQ plots. We expect normal distributions in most of the variables defined as raw scores from appropriate tests, or in their log transformations (e.g., tests measuring time needed to walk certain distance or to perform a task). The groups will be compared in their characteristics, primary, and secondary outcomes using the one-way analysis of variance (ANOVA), and its non-parametric version (Kruskal—Wallis test), where needed. The Benjamini-Hochberg correction (102) will be used to account for multiple comparisons. We expect no differences between groups on the baseline. If differences are observed, these will be accounted for in the longitudinal models.

#### Measuring the effectiveness of rehabilitation

To test hypothesis I., i.e., a positive influence on all outcomes, the effect of therapy for each therapeutic group will be assessed separately by paired *t*-tests performed on pre-test and post-test scores. Two sample *t*-test will be used to compare the patients in the three COMIRESTROKE groups with patients from the control group. The Wilcoxon signed-rank test or Mann-Whitney test will be used on data where normal distribution cannot be expected.

To test hypotheses II. and III, a comparison between therapeutic groups will be performed using two sample Student *t*-tests on differences between measurement time points (or Mann-Whitney tests, respectively, for data, where normal distribution of the differences cannot be expected). For hypothesis II, the COMIRESTROKE—ICF group will be compared against the participants in other three COMIRESTROKE groups (NEFI, TECH and CONTROL). For hypothesis III, the COMIRESTROKE—NEFI group will be compared against the other three COMIRESTROKE groups (ICF, TECH and CONTROL). A one-sided alternative will be tested according to our hypotheses. We will also implement the one-way ANOVA test to jointly compare all four COMIRESTROKE groups for pre-post differences. In addition, a Tukey *post-hoc* comparison will be made to detect any further group differences beyond our hypotheses.

To assess the overall impact of rehabilitation and to compare effectiveness of different therapeutic approaches for Hypotheses I—III in more complex way, and to test for effect of other covariates in Hypothesis V, a linear mixed effect model will be used with random patient effect fitted to longitudinal patient data. Measurement effect, group effect and their interaction, as well as effects of other covariates such as the level of disability in admission time will be tested by *F*-test.

To address Hypothesis IV and Exploratory Goal I, correlations between changes in different examination scores will be evaluated using Pearson correlation coefficient and its non-parametric analogies. A cluster analysis will be performed to identify different phenotypes. This will allow the identification of different groups of patients in relation to the efficacy of the neuro-rehabilitation programs.

To address Goal II, for selected multi-item instruments, item-response theory (IRT) and generalized linear regression models will be used to study differential item functioning with respect to rehabilitation groups. Account will be taken for other respondent characteristics. Use will be made of differential item functioning in change (DIF-C) analysis (7) in order to detect between-group differences.

#### Statistical software

Analysis will be performed using the free statistical environment R (103) and its libraries. The lme (104) and nlme (105) library will be used for implementing mixed effect models. Library difNLR (106) will be used for the detection of DIF. Modules of the interactive ShinyItemAnalysis application will enable lme (104), nlme (105) and difNLR (106) library sample analyses to be interactively displayed (107).

#### Data monitoring

Data quality will be monitored by an independent person within 2 days after each set of assessments (pre, post, follow-up) is completed. An interim data analysis will be conducted by an independent data analyst each month or after data from at least 10 new patients are collected. The trial will be terminated when planned number of participants (*n* = 280) is collected.

#### Harms

This study poses no greater risks than standard care. Examinations will be carried out by competent examiners and therapy by qualified specialists. The program might be customized to prevent patient's exertion, although that is not anticipated, since the level of exercise load should be mild.

We do not expect adverse events which could cause trial termination. Any potential adverse events will be reported in https://register.clinicaltrials.gov.

### Article summary

#### Strengths and limitations of this study

- This Four-Arm Parallel-Group Randomized Double Blinded Controlled Trial with a longitudinal design will provide evidence of the effectiveness of COMprehensive Intensive REhabilitation Program after STROKE (COMIRESTROKE) on a wide range of primary and secondary outcomes, and long non-coding RNA readouts.
- Comparing different kinds of COMIRESTROKE will provide evidence for on optimal approach to ischemic stroke rehabilitation.
- Blood sampling will assist with offering initial evidence that lncRNAs represent molecular readouts of effective rehabilitation and will provide information about the direct/indirect role of lncRNA in brain neuronal protection before and after COMIRESTROKE.
- The limitation of this study is the difficulty in blinding the treatment from patients.
- Looking for tools regarding the interdisciplinary team management in inpatient care after stroke as well for further feedback, the team has chosen the ICF model and its tools for this purpose. The authors are aware that the ICF model can be used in interdisciplinary, multidisciplinary as well as single-discipline rehab approaches. Coupling the ICF tools with the interdisciplinary rehabilitation management approach in this study is inserting a confounding factor since there may be differences in the effectiveness of interdisciplinary vs. multidisciplinary irrespective of the ICF. This effect must be further studied in future projects comparing the multidisciplinary and interdisciplinary team management approach with/without the ICF tools.
- It is a very large study, the implementation of which depends on good organization of the whole team, both in the neurology and rehabilitation clinics, so control mechanisms must be put in place to avoid data loss.
- A wide range of patient characteristics will be collected. To avoid overburdening patients, the examination will be effectively organized over 2 days. Additionally, examination by a clinical psychologist and speech therapist will be done in such a way as to bring a therapeutic effect.
- One of the most important outputs of this clinical trial will be the recommendation concerning which clinical tests and scales are the most useful in post-stroke rehabilitation based on the framework of the ICF model. This finding will only apply to tests selected for our protocol that was influenced by recommendation of Czech Ministry of Health, and as such may not be applicable generally.
- As in clinical practice, multi-item measurements are used to guide rehabilitation programs, we consider that an item-level analysis may provide a deeper insight into the effectiveness of different interventions.
- The development of the protocol was influenced by the health care organization in the Czech Republic where multidisciplinary team management prevails and PT category neuroproprioceptive “facilitation, inhibition” is predominantly used/applied. Therefore, the results of the study may not be applicable in other healthcare systems.
- In conclusion, the proposed study will determine the effectiveness of COMprehensive Intensive REhabilitation Programs after STROKE involving various approaches of management (interdisciplinary team work implementing individualized rehabilitation using the International Classification of Functioning, disability and health model as a guide with multidisciplinary team work) and physical therapy categories (neuroproprioceptive “facilitation and inhibition” physical therapy and motor/skill acquisition implementing technology-based physical therapy) *via* analysis of primary, secondary and lncRNA readouts.
- This clinical trial will offer an innovative approach not previously tested and will provide new complex analysis along with public assessable molecular biological evidence of various rehabilitation methodologies for the alleviation of the effects of ischemic stroke.

#### Ethics and dissemination

The Ethical committee of the Institute for the Clinical and Experimental Medicine and Thomayer University Hospital have approved the study under number 09306/22 (6-21_01). Written, informed consent to participate and provide blood samples will be obtained from all participants (information for participants and inform consent are available at in https://register.clinicaltrials.gov). Protocol amendments are not expected, but in case that any amendments are needed, such amendments will be subject to approval by The Ethical committee of the Institute for the Clinical and Experimental Medicine and Thomayer University Hospital.

Personal information will be collected, shared, and maintained in order to protect confidentiality according to valid laws of the Czech Republic before, during, and after the trial. Access to the dataset will be given to independent data analysts and will be limited for investigators. Participation in the study has a minimum of known risks, however, in the event of any damage to the study participant related to his/her participation in the project, this person will be compensated by hospital insurance. Study results will be published in journal articles and reported on https://register.clinicaltrials.gov. Anonymized data will be disseminated *via* https://osf.io or other data sharing platforms, anonymized lncRNA expression data will be uploaded onto Genbank and made freely available to the scientific community.

The current approved version of the protocol NCT05323916 is version released 04/27/2022. The trial has yet not commenced. First patient enrolment is planned for May 2022. Recruitment will be completed by December 2025.

## Ethics statement

The studies involving human participants were reviewed and approved by the Ethical Committee of the Institute for the Clinical and Experimental Medicine and Thomayer University Hospital. The patients/participants provided their written informed consent to participate in this study.

## Author contributions

KŘ, PM, MP, BM, and TP: conceptualization. KŘ and TP: resources. KŘ, VO'L, and PM: writing-original draft preparation. KŘ, PM, MV, BM, MP, JHl, DH, ŠP, MN, KP, PB, RV, JHa, VO'L, MČ, AB, and TP: writing—review and editing. KŘ, PM, KP, and JHa: project administration. All authors: methodology and electronic case report form. All authors have read and agreed to the published version of the manuscript.

## Funding

Ministry of Health, Czech Republic – RVO (Thomayer University Hospital – FTN, 00064190) supported the development of the design of the study, including payment of license fees and data recording system. PM and MV were supported by the long-term strategic development financing of the Institute of Computer Science (RVO:67985807). JH was supported by the grant no. SGS20/167/OHK3/3T/13 of the Czech Technical University in Prague. The research was also supported by Charles University, programme Cooperatio (Neuroscience and Medical Diagnostics and Basic Medical Sciences, the field ‘Medical Genetics’) and 260533/SVV/2022.

## Conflict of interest

The authors declare that the research was conducted in the absence of any commercial or financial relationships that could be construed as a potential conflict of interest.

## Publisher's note

All claims expressed in this article are solely those of the authors and do not necessarily represent those of their affiliated organizations, or those of the publisher, the editors and the reviewers. Any product that may be evaluated in this article, or claim that may be made by its manufacturer, is not guaranteed or endorsed by the publisher.
